# Reduced learning rates but successful learning of a coordinated rhythmic movement by older adults

**DOI:** 10.1177/17470218241240983

**Published:** 2024-04-12

**Authors:** Daniel Leach, Zoe Kolokotroni, Andrew D Wilson

**Affiliations:** Psychology, School of Humanities and Social Sciences, Leeds Beckett University, Leeds, UK

**Keywords:** Coordinated rhythmic movement, learning, transfer, ageing

## Abstract

Previous work has investigated the information-based mechanism for learning and transfer of learning in coordinated rhythmic movements. In those papers, we trained young adults to produce either 90° or 60° and showed in both cases that learning entailed learning to use relative position as information for the relative phase. This variable then supported transfer of learning to untrained coordinations +/30° on either side. In this article, we replicate the 90° study with younger adults and extend it by training older adults (aged between 55 and 65 years). Other work has revealed a steep decline in learning rate around this age, and no follow-up study has been able to successfully train older adults to perform a novel coordination. We used a more intensive training paradigm and showed that while older adult learning rates remain about half that of younger adults, given time they are able to acquire the new coordination. They also learn to use relative position, and consequently show the same pattern of transfer. We discuss implications for attempts to model the process of learning in this task.

## Introduction

This article is part of a series of studies designed to investigate the perception–action mechanisms supporting learning and transfer of learning in coordinated rhythmic movements. It is a direct replication and then extension of the work of Leach et al. (2021b). That study extensively trained young adult (YA) participants to produce a 90° pattern and measured both learning and transfer of that learning. Participants learned to produce 90° stably by learning to perceive it via relative position information (Wilson & Bingham, 2008), and this learning transferred substantially to 60° and 120°. This was the first time such transfer had been observed using this task. In the current study, we performed a direct replication of this study with younger adults, and then extended the task to investigate perception–action learning in a group of older adults ([OAs] 56–63 years old). Previous work (Coats et al., 2013, 2014; Ren et al., 2015) had shown a large drop in learning rate around this age, which resulted in OAs failing to improve at the task.

We had two goals in this article. The first was to replicate the results of Leach et al. (2021b) with younger adults, which we did very robustly. Second, we wished to investigate whether the more extensive and learner-driven training protocol we used would provide enough time for OAs to acquire 90°; we could then look to see whether they showed the same patterns of transfer and the switch to relative position as younger adults. Broadly, the answer was “yes”; OAs successfully learned to produce 90°, they did so by learning to perceive it via relative position, the learning transferred to 60° and 120°, and on average these results were the same as for the younger adults. There were age group differences in exactly how they progressed through the training, however, that have some useful implications for future research on how age affects perception–action learning.

### Coordinated rhythmic movement

Coordinated rhythmic movement is a useful lab-based task for studying the mechanisms of perception–action learning. The dynamics of performance and learning have been extensively studied (see Kelso, 1995 for an overview) as have the underlying perception–action components (see Golonka & Wilson, 2012, 2019, and Wilson, 2022 for reviews), and these perception–action dynamics have been mechanistically modelled (Bingham, 2001, 2004a, 2004b; Herth et al., 2021; Snapp-Childs et al., 2011).

Prior to training, only mean relative phases of 0° and 180° can be produced stably; other coordinations are not stable, with 90° being the least stable. These other relative phases can be trained but this training requires some form of augmented feedback. At higher frequencies, movements become less stable and there is a tendency to transition from 180° to 0°, but not the other way around. This pattern persists if the coordination is being produced bimanually by one person (e.g., Kelso, 1984), unimanually between two people (e.g., Schmidt et al., 1990), or unimanually between a person and a display (e.g., Wimmers et al., 1992). They also persist in perceptual judgements of coordinated rhythmic movement, with no movements generated by the observer (e.g., Zaal et al., 2000). Bingham’s perception–action model (Bingham, 2001, 2004a, 2004b; Snapp-Childs et al., 2011) explains all these effects as emerging from the activity of a perception–action dynamical system, in which two oscillators are coupled together via perceived relative phase. The model proposes that the relative phase is perceived via the relative direction of motion, the discrimination of which is modified by the relative speed. Relative direction is most stable at 0°, moderately stable at 180°, and maximally unstable at 90°, and this perceptual fact is what establishes the patterns described above. The model has received extensive empirical support (reviewed in detail in Golonka & Wilson, 2019).

Relative direction is maximally variable at 90°, and so even if it were clearly detected at this relative phase (which it is not), it would not be of much use for learning to produce stable actions there. However, it is possible to learn 90° (and other coordinations) because other useful information variables about relative phase are present during a coordinated rhythmic movement. Which variable is being used can be identified via a perturbation method, in which candidate information variables are selectively made uninformative about relative phase. Wilson and Bingham (2008) did this testing and confirmed that learning to perceive 90° entailed switching to the relative position. Leach et al. (2021b) then confirmed with perturbation methods that learning to move at 90° also entailed switching to the relative position.

Herth et al. (2021) developed an extension of the Bingham model to account for learning 90°. The model proposes that participants initially use relative direction to perceive when they are moving in the ballpark of 90°. Relative position information about 90° then becomes present in the displays, and participants begin to differentiate and use it, with increasing success (as per the ecological *direct learning* framework; Jacobs & Michaels, 2007).^1^ The model contains a threshold-based mechanism that dynamically switches between the relative direction and relative position drivers once the latter has been learned, and can model both learning and trained performance.

Finally, learning relative position also accounts for observed transfer of learning in this task. Learning to clearly perceive 90° allows stable movement at 90° (Wilson, Snapp-Childs, & Bingham, 2010), and learning to move stably at 90° leads to improved perceptual thresholds at 90°; Snapp-Childs et al. (2015) showed this and transfer between unimanual and bimanual versions of the task, while Leach et al. (2021b) showed this and transfer from 90° to 60° and 120°. Leach et al. (2021a) then confirmed that learning 60° entailed learning relative position as well, which led to transfer to 90° and (unexpectedly^2^) 30°. The pattern of how learning transfers in this task is explained by the fact that people learn relative position, and that relative position supports the perception of relative phase across a variety of relative phases. How learning transfers therefore is another way of measuring what has changed with learning.

### Coordination learning and ageing

As a general rule, ageing reduces the ability to acquire new skilled actions (Voelcker-Rehage, 2008). This usually shows itself as a reduction in the learning rate; OAs are slower to learn a new skill. This reduction is task-specific, however (e.g., Leversen et al., 2012), and so the exact mechanisms behind the reduction are not yet clear. This reduced skill acquisition has real-world consequences; for example, strokes are more common in OAs and successful rehabilitation often involves the acquisition or re-acquisition of skilled movements (Cancelli et al., 2011). It is therefore worth investigating what causes the problems, and ways to support OAs in skill acquisition. As laid out above, coordinated rhythmic movement is a key task for studying and modelling perception–action mechanisms of skill acquisition and skilled performance, and it has therefore been used to investigate changes to this mechanism with ageing. We will focus here on work about learning new coordinations.

Swinnen et al. (1998) and Wishart et al. (2002) had YA and OAs learn a 90° coordination, and showed that OAs were slower to learn and were more variable in their performance. A limitation of this work, however, is that it relied on Lissajous feedback. These displays take the two sinusoidal movement trajectories of the limbs being coordinated, and graph them in a two-dimensional (2D) display, one trajectory per axis. 0° produces a straight line of Slope 1, and 180° has Slope −1. 90° produces a circle, and all other relative phases produce ellipses of varying eccentricity. This feedback has been used in a number of studies when training coordinated rhythmic movements such as 90°; these coordinations are unstable because they are poorly perceptually discriminated (Zaal et al., 2000) and people therefore need help to bootstrap their way into improving this discrimination. However, Lissajous displays *transform* the visual information about the coordination and as such it changes the underlying perception–action task dynamics (specifically, relative direction and relative position are not defined in a Lissajous display). This new dynamic shows simpler patterns of behaviour: typically unstable relative phases such as 90° become relatively straightforward with just a little practice (Kovacs et al., 2009). This performance depends on the presence of the Lissajous feedback, however. This all suggests that with Lissajous feedback, people are not so much learning novel coordinations as they are learning a simpler perception–action coupling to the augmented feedback, which is fine except that it means none of the perception–action models of the coordination task apply anymore, which makes it harder to interpret the results in terms of changes to the perception–action mechanism.

Wilson, Snapp-Childs, Coates, & Bingham, (2010) solved these issues by developing a feedback method that does not transform the visual information: *coordination feedback*. These displays preserve the movement kinematics of the limbs being coordinated and simply change the colour of the display when the person is producing the target relative phase, ± a customisable error bandwidth which we usually set to be initially large (e.g., 30°) and then fade over time. This serves as a neutral cue that helps constrain the person to produce movements that spend longer in the ballpark of the target relative phase, and the fading helps drive the person to continue to improve. Then, because the displays are spending more and more time showing the target relative phase, this provides the person with more and more time to learn to differentiate the relative position information about that relative phase which they can then use to produce that target on their own. Wilson et al. showed that (a) feedback is required for people to acquire a novel coordination, (b) people can use coordination feedback to improve their performance, and (c) they do not become reliant on the presence of the feedback, suggesting that it has not altered the task dynamic. This feedback has been successfully used in a variety of experiments on learning (Bingham et al., 2018; Herth et al., 2021; Huang et al., 2019; Leach et al., 2021a, 2021b; Snapp-Childs et al., 2015; Zhu et al., 2017).

Coordination feedback displays have also been used to study perception–action learning and changes with ageing. Coats et al. (2013) trained three groups of participants aged in their twenties, seventies, and eighties to produce 90°. The two OA groups improved much less than the YAs, and showed a learning rate of about half. Coats et al. (2014) then trained seven groups to produce 90° (ages in the twenties, thirties, forties, fifties, sixties, seventies, and eighties). They showed that the drop in learning rate was abrupt, not gradual, and occurred between the fifties and sixties groups (hence called “the 50s cliff”).

Both of these studies relied on visual feedback displays, and there are known decreases in the ability to visually discriminate motion information (reviewed in Andersen, 2012). Coats et al. (2013, 2014) therefore proposed that the problem was being caused by a decreased ability to visually discriminate the motion information (relative position) required to learn 90°. To test this, Ren et al. (2015) trained YAs (twenties) and OAs (fifties, sixties, seventies) using either visual or haptic feedback. If the problem is visual, then haptic training should bypass the problem. This had no effect, however; learning rates plummeted the same way in both visual and haptic training groups, again between the fifties and sixties. Ren et al. concluded that the problem is not visual, nor haptic, but a perception–action problem: OAs are less able to learn either the information, or how to couple information to an action.

There are two possible places for ageing to have an effect in the process of learning as modelled in Herth et al. (2021). First, OAs may have trouble learning to differentiate relative position and thus must continue to rely on the feedback plus (variable) relative direction information. Because no-one has yet successfully trained OAs to produce 90°, no-one has been able to explicitly test if they have begun using relative position via perturbation methods. The current study tested all participants’ post-training with the perturbation task to explicitly identify the information variable being used to perceive 90°, and will also look to see if the OAs show the same pattern of transfer of learning underpinned by acquiring relative position (Leach et al., 2021b). Second, and more likely at this point, OAs may simply be slower at moving through the process and they have not yet been given enough time to show what they can learn. The OA work cited above has used 50–60 training trials in total, and while this enough for younger adults, given the observed learning rate difference it is likely not enough for the OAs. This study instead uses the performance-based training criteria for establishing whether an individual has learned the task developed in Leach et al. (2021a, 2021b) and allows for participants to experience up to 300 training trials if required.

### The current study

This study trained a group of younger adults (in their twenties) and a group of OAs (aged between 55 and 65 years, to straddle the “50s cliff”) to produce bimanual coordinated rhythmic movements at 90°, using coordination feedback and a more intensive training schedule than the previous work cited above. We assessed coordination stability at 0°, 30°, 60°, 90°, 120°, 150°, and 180° before and after training and at retention to measure learning and transfer of learning. We also measured perceptual judgements of 90° in these sessions, using both unperturbed displays (to measure thresholds) and position perturbation displays (to identify whether people were now perceiving 90° via relative position; Wilson & Bingham, 2008). We predicted^3^ that we would fully replicate the younger adult results from Leach et al., 2021b, and that the extended training would allow OAs to learn 90° (although perhaps still not to the same extent as the YAs). We also predicted that the OAs would improve at 90° by acquiring relative position, measured via perturbation methods and the pattern of transfer, supporting the hypothesis that they are learning in the same way, just slower.

## Method

### Transparency and openness

This experiment’s design and analysis plan was preregistered (Leach et al., 2019; young adult study, https://osf.io/x4nef; older adult study, https://osf.io/q3k5e). Data and analysis files are available at https://osf.io/qvzuh/.

Note that gaze data were collected using a Tobii TX300 eye tracker; however, these data will not be analysed here. The eye tracker is screen mounted; no chin rest is required and participant movement is unrestricted (within a fairly comfortable range) and so presenting the task on this system is effectively identical to the previous experiments (Leach et al., 2021a, 2021b). This study is therefore an exact replication (YAs) and extension (OAs) of that of Leach et al. (2021b).

### Participants

Ten YAs participated in this study, one of whom chose not to complete the entire procedure leaving a total of 9 participants (18–25 years old, *M* = 19.8; male = 4, female = 5). Seven OAs participated, two of whom chose not to complete the entire procedure leaving a total of 5 participants (56–63 years old, *M* = 60.2; all female). Recruitment was guided by a power analysis based on Leach et al. (2021b; see the Online Supplementary Material 1). Data were collected in 2019.

All participants were free from known neurological defects or motor disabilities, had normal or corrected-to-normal vision, and were right-handed (measured with the Edinburgh Handedness Inventory; Dragovic, 2004; Oldfield, 1971). All participants were naïve to the experimental questions. Prior to training, all participants’ relative phase production matched the predefined criterion for participation (see *Criteria*). All participants were recruited using a convenience sample in the surrounding area of Leeds, UK; YAs were paid £15 upon competition of the study, while the OAs were paid £50.^4^ Ethical approval was granted by the Psychology Ethics Committee at Leeds Beckett University, UK.

### Design

The design was identical to that used in Leach et al. (2021b); all participants were assigned to learn a 90° relative phase. All participants performed two types of experimental task: coordinated rhythmic movements *(Action)* and two-alternative forced choice *(Judgements)*.

For the Action tasks, there were two within-subject variables. The first was Session (3 levels; Baseline, Post-training, Retention). These sessions were referred to as Assessment sessions, to distinguish them from the Training sessions. The second was Target phase (7 levels; 0°, 30°, 60°, 90°, 120°, 150°, and 180°). The dependent variable was the Proportion of Time on Target phase ±20° (PTT20), a valid measure of performance (Wilson, Snapp-Childs, Coats, & Bingham, 2010).

For Judgement tasks, there was one within-subjects variable, Session (3 levels; Baseline, Post-training, Retention). The dependent variable was the estimated threshold to identify 90° in the Judgement tasks (the lower the threshold, the greater the ability to discriminate 90°).

### Materials

All sessions were performed on a Windows PC with a 520 × 285 mm Tobii TX300 monitor located approximately 70 cm from the participants. The computer presented a display of two white dots (~15 mm), separated vertically (~35 mm), that moved horizontally across a black background (screen refresh rate 60 Hz, resolution 1,920 × 1,080). The motion of both dots was centred at the screen centre with an amplitude of 300 pixels (~115 mm). All displays were presented, controlled, and recorded by a custom MATLAB toolbox written by ADW incorporating the Pyschtoolbox (Kleiner et al., 2007; http://psychtoolbox.org). MATLAB 2014b was used to record and analyse the data.

**Figure 1.**
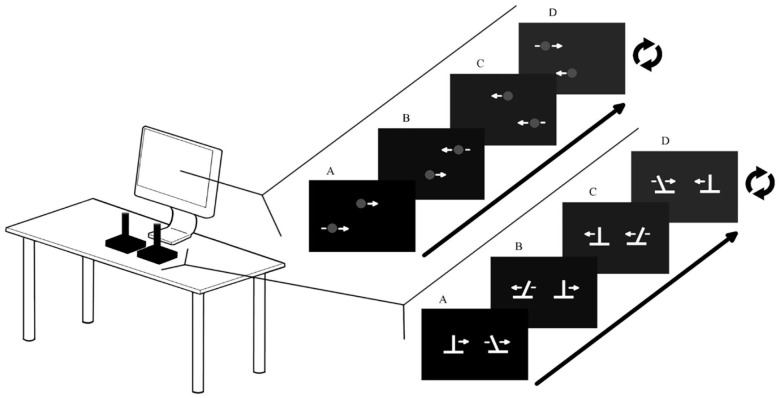
Experimental set-up: Action sessions. Participants use both joysticks to control the horizontal movements of the dots on the computer display. The visual display on the computer screen (A) corresponds with the position of the joysticks (A). The figure shows an example of moving at 90°. This is achieved by moving linearly from A to D and repeating. During the training sessions, moving at 90° ± some error triggers the hot–cold signal in which the white dots turn green (grey in figure).

For Action sessions, participants used two USB Logitech Extreme 3D Pro joysticks. The central spring and the rubber guard were removed to disable force feedback (see Figure 1). The vertical position of both dots on the screen was fixed, but the horizontal position of both dots was controlled by the horizontal position of the joysticks, with the left and right joystick corresponding to the top and bottom dots, respectively. The mapping of the joysticks to screen amplitude is set so that the required amplitude on the screen does not entail hitting the limits of the joystick range of movement. This forces participants to actively control the joysticks as much as possible, rather than to simply slam into the joystick endpoint to stop.

For Judgement sessions, the participant responded to displays using a USB keyboard, responding with the “A” and “L” keys for the first and second choice, respectively.

### Procedure

Participants performed between 9 and 13 separate sessions on separate days (see Table 1). The exact number of sessions performed by each individual participant was dependent on when various criteria were met during training (see *Criteria*). During the Baseline assessment session, participants performed three different tasks (two Action, one Judgement) in the order described (approximately 45 min to complete). In the Post-training and Retention assessment sessions, participants repeated the procedure from Baseline with one additional perturbation Judgement session described below (approximately 60 min to complete). Participants completed the Baseline, Training, and Post-training sessions within a 3-week time frame, and completed the Retention session 14–24 days after the Post-training session. Each Training session took approximately 20 min to complete.

**Table 1.** Experimental design.

Baseline *1 Session*	5 × 20 s trials each of bimanual 0°, 180°, *assigned phase* (90°) *Criterion for participation: 90°* < 0*° & 180°; assigned phase (90°)* *<* .*50* 5 × 20 s trials each of bimanual 30°, 60°, 120°, 150°
	2AFC judgement task (*assigned phase*, 90°)
Training	30 × 20 s trials bimanual 90° w/feedback ± 30°
	30 × 20 s trials bimanual 90° w/feedback ± 25°
7–10 sessions	30 × 20 s trials bimanual 90° w/feedback ± 20°
	30 × 20 s trials bimanual 90° w/feedback ± 15°
	30 × 20 s trials bimanual 90° w/feedback ± 10° 30 × 20 s trials bimanual 90° w/feedback ± 10°
Post-training	5 × 20 s trials each of bimanual 0°, 180°, *assigned phase* (90°) 5 × 20 s trials each of bimanual 30°, 60°, 120°, 150°
1 session	2AFC judgement task (*assigned phase*, 90°) 2AFC judgement task (Perturb Position, *assigned phase*, 90°)
Retention 1 session	5 × 20 s trials each of bimanual 0°, 180°, *assigned phase* (90°) 5 × 20 s trials each of bimanual 30°, 60°, 120°, 150° 2AFC judgement task (*assigned phase*, 90°) 2AFC judgement task (Perturb Position, *assigned phase*, 90°)

All participants worked through these tasks in the order noted. The feedback bandwidth (e.g., ±30°) indicates over what range from the target-phase the colour feedback is triggered. This is faded over time to drive learning (Wilson, Snapp-Childs, & Bingham, 2010; Wilson, Snapp-Childs, Coates, & Bingham, 2010). See Criteria regarding the performance-based progression employed.

#### Action task (assessment sessions)

All participants were shown an 8-s, 1 Hz demonstration of the first target relative phase (0°) and performed one 20-s practice trial of producing that relative phase at 1 Hz with the joysticks. Participants then performed one block of four 20-s trials in which they controlled the horizontal motion of both dots. The top dot was controlled by the left hand, the bottom dot by the right hand. Participants were instructed to move the joysticks in a smooth, side-to-side, movement to produce the target-phase at 1 Hz. This block structure was then repeated for 180° and 90° relative phase, in that order.

These data were used to ensure that none of the participants were already able to perform 90° at a level equivalent to 0° and 180° and could take part in the study (see Criteria). After this, participants performed a second set of coordinated rhythmic movements to measure baseline performance at 30°, 60°, 120°, and 150°, using the same structure as above.

#### Judgement task

Following the action tasks, participants performed a series of two-alternative forced choice (2AFC) judgements for 90°. 2AFC is a standardised psychophysical measure for determining perceptual thresholds (see Leach et al., 2021a, 2021; Snapp-Childs et al., 2015; Wilson & Bingham, 2008; Wilson et al., 2010 for applications to coordination perception).

Each trial started with a 4-s demonstration trial of 90°, followed by the presentation of a pair of successive displays. Both displays contained two dots moving harmonically on the screen at some mean relative phase, for 4 s at 1 Hz. The dots were centred on the screen, with an amplitude of 300 pixels (~11.5 cm). Of each pair, one showed two dots moving at 90°, and the other was different from 90°; the order was randomly selected on each trial. The task for the participants is to choose which one of the displays shows 90° (pressing “A” for the first and “L” for the second, with no speed requirement).

How different the two displays were was determined using two independent but interleaved transformed 1-up/2-down staircase procedures. One staircase controlled the different displays less than 90°, one for those greater than 90°. Both used a step size “up” of 10° and a stop rule of 8 reversals. Step size “down” was fixed to 54.88% of the step size “up” according to Table 5.1 of Kingdom and Prins (2009); here 5.48°. The initial difference for each staircase was set to 30° and trials stepped down only until the first reversal (first error), after which the staircase procedure was applied. Participants are given knowledge-of-results feedback after each trial (“Correct!” or “Incorrect!”).

In the Post-training and Retention sessions, participants repeated the 2AFC task and then completed an additional 2AFC task in which a *position perturbation* is applied to the display (Wilson & Bingham, 2008). In these displays, the amplitude of the top dot is changed at random on every half-cycle, with the constraints that the dot must cross the midline of the screen and cannot exit the screen. The amplitude of the bottom dot is then set to half the top dot’s amplitude, so that it varies randomly but in a way that is coupled to the other dot—this preserves the relative phase. Where and when peak amplitude and peak velocity occur therefore change on every half-cycle. This preserves mean relative phase (and relative direction information about that relative phase) while making it impossible to use relative position information to perceive relative phase, because there is no stable information about where the dots are within their cycles. This perturbation tests the hypothesis that learning to improve at 90° entails switching to using relative position.

#### Action task (training)

Following Baseline assessment, participants were trained to bimanually produce 90°. The number of training sessions completed by each participant depended on their performance (see *Criteria)*.

During each training session, participants performed 30, 20-s trials where their goal was to produce 90°. Participants received *coordination feedback* (Wilson, Snapp-Childs, Coats, Bingham, 2010) for all trials except for every fifth trial (this colour feedback was not present in the Assessment Action tasks; for that reason, coordination feedback is removed every fifth trial to help prevent dependence on it: Kovacs et al., 2009; Snapp-Childs et al., 2015). This feedback changed the colour of the dots from white to green when performance was within the given error bandwidth of the target relative phase. In the first training session the error bandwidth is set at ±30° and was reduced by ±5° across sessions when the Criterion for Progression was met (to ±25°, ±20°, ±15°, ±10°).

After every trial with feedback, participants also received knowledge-of-results feedback based on their performance, in which the participant is given a performance percentage (their PTT20 score as a percentage) and a comment (see Table 2). Finally, participants received additional knowledge-of-results at the end of each training session in the form of a level-progression statement. This simply stated whether or not the participant would stay at the current level or progress to the next level. We found that this helped participants stay on task and remain motivated through the extensive training.

**Table 2.** Knowledge of results (performance generated score).

Performance	Comment
<25%	*=* *This is still a little low—keep trying!*
25%–50%	*=* *Definitely improving—keep it up!*
50%–75%	*=* *Doing great—keep it up!*
>75%	*=* *This is really great—great job!*

##### Criteria

Prior to training, all participants’ 90° production was substantially worse than 0° and 180° (YAs, mean PTT20: 0.20, 0.77, 0.77, respectively; OAs, mean PTT20 0.14, 0.69, 0.72, respectively). Participants were then trained in accordance with several predefined criteria. In each training session, when PTT20 was greater than 0.5 in at least 20/30 trials, the participant progressed to the next training stage. This was used to confirm that the participant was ready for progression and to avoid occasional poor performance trials from halting progression. Meeting this criterion resulted in the feedback bandwidth of the next training session to be reduced by ±5°; otherwise the feedback was kept the same. Training was stopped if PTT20 was greater than 0.6 in at least 20 trials for the last two training sessions (feedback bandwidth at ±10°), or when participants completed 10 training sessions. The number of training sessions across YA participants varied between 7 and 10, and all but 2 YA participants progressed to and completed at least one session with the feedback bandwidth set to ±10°. OA participants all used all 10 sessions, and only one made it to ±10°.

#### Data analysis

##### Judgements

For the judgement tasks, the computer recorded the responses (“correct” or “incorrect”) in relation to the relative phase of the “different” displays that were shown. We separately averaged the difference from 90° of relative phases at which reversals in the staircase procedure occurred for the “different” phases that were greater than 90° and those less than 90°, excluding the first reversal, for each participant. We then averaged those thresholds for each participant.

##### Movement

The raw movement data is a 60-Hz time series of the position of the joysticks over time. Each time series was centred on 0, filtered with a low-pass Butterworth filter (cut-off frequency 10 Hz), and differentiated to compute the velocity time series. The continuous phase time-series of each joystick was computed as the arctan(V/X) for each data point and the difference between these time series was the relative phase time series. We then computed the proportion of this time series that fell within 20° of the target relative phase (PTT20).

##### Contrast analyses

To analyse transfer of learning we used dependent measures contrast analyses (Rosenthal et al., 2000). This analysis allows us to test for a specific hypothesised pattern of differences across multiple means with a single test (rather than the less powerful and less targeted method of an ANOVA followed by pairwise comparisons). In this experiment, we applied a contrast analysis to performance in the three Assessment sessions at each untrained relative phase in which we tested for the specific pattern of change observed at the trained relative phase of 90°.

The test statistic, *t*, is computed as

Equation 1. $$t_{contrast}=\frac{\bar{L}}{\sqrt{\frac{{\hat{\sigma }}_{L}^{2}}{n}}}\mathrm{with}L_{i}=\sum_{j}^{k}(x_{ij}\cdot \lambda_{j})$$

where *x* is the data and λ are weights. The λ weights are the way of quantifying the hypothesised pattern, here set by Assessment session performance at 90° (see below). If the data do not differ in the specific way implemented by the Lambda weights (λ), then *L*(*i*) is near to zero, that is, *H*_0_ is *L*(*j*) = 0. In terms of transfer, a statistically significant *L*(*i*) score for data at a particular untrained relative phase indicates that the specific pattern of improvement and retention observed at 90° is also occurring at that particular untrained phase; the learning has transferred.

## Results

We will first examine the Younger Adult data as a direct replication of that of Leach et al. (2021b). We will then examine the OA data, as an extension of Leach et al. (2021b). The structure of these analyses will follow Leach et al. (2021a, 2021b). Finally, we will make some direct comparisons between the YA and OA data. All these analyses were preregistered (Leach et al., 2019; young adult study, https://osf.io/x4nef; older adult study, https://osf.io/q3k5e).

### YAs

#### Action task

##### Learning

**Figure 2.**
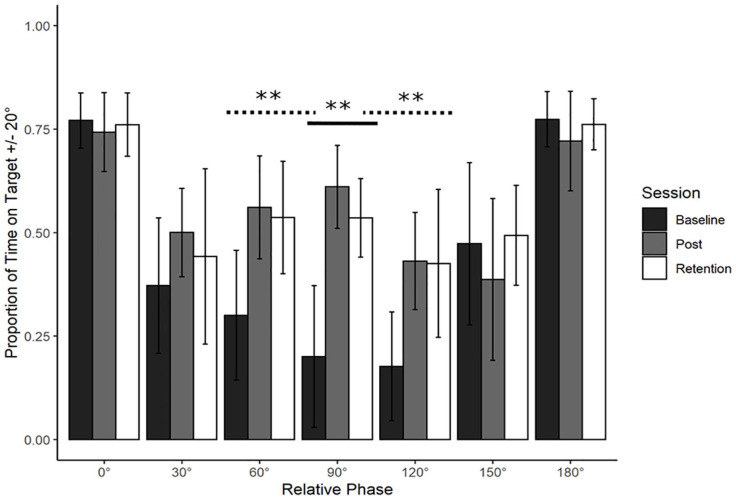
Young adults trained at 90°: Average action data. Average performance data (Proportion of Time on Target ±20°) with standard error bars for all phases in the three assessment sessions (Baseline, Post-training and Retention). Significance levels are indicated on the figure (** = *p* < .01). There was a significant main effect of Session for the trained phase of 90° (solid line). This learning transferred to 60° and 120° (dotted lines, see “Transfer” section for further details).

Refer to Figure 2. To examine whether and how training at 90° changed performance at 90°, average PTT20 was analysed using a one-way repeated measures ANOVA with Session (Baseline, Post-training, Retention) as a within-subject factor. Descriptive statistics show a difference between Baseline (*M* = 0.20, *SD* = 0.17), Post-training (*M* = 0.61, *SD* = 0.1), and Retention (*M* = 0.54, *SD* = 0.095).

YA participants significantly improved their coordination stability at 90° from Baseline to Post-training and that learning was partly retained. There was a significant main effect of Session, Greenhouse–Geisser corrected *F*(1.13, 9.04) = 60.38, *p* < .001. Bonferroni-adjusted post hoc analyses revealed a significant difference between Baseline and Post-training, *t*(8) = −8.617, *p* < .001, *MD* = −0.41, Baseline and Retention, *t*(8) = −7.015, *p* < .001, *MD* = −0.335, and between Post-training and Retention, *t*(8) = 5.417, *p* < .01, *MD* = 0.075. Production of 90° was poor at Baseline, it improved significantly with training and this improvement decreased slightly after the retention period.

##### Transfer

The learning analysis established the pattern of learning and retention at 90° that we will look for at other relative phases, to measure transfer of learning. For the contrast analysis we use here, the λ weights were set at −3 for Baseline, 2 for Post-training and 1 for Retention, in accordance with the guidelines set by Rosenthal et al. (2000). We show transfer of learning with a significant contrast analysis using these weights at a given relative phase.

**Figure 3.**
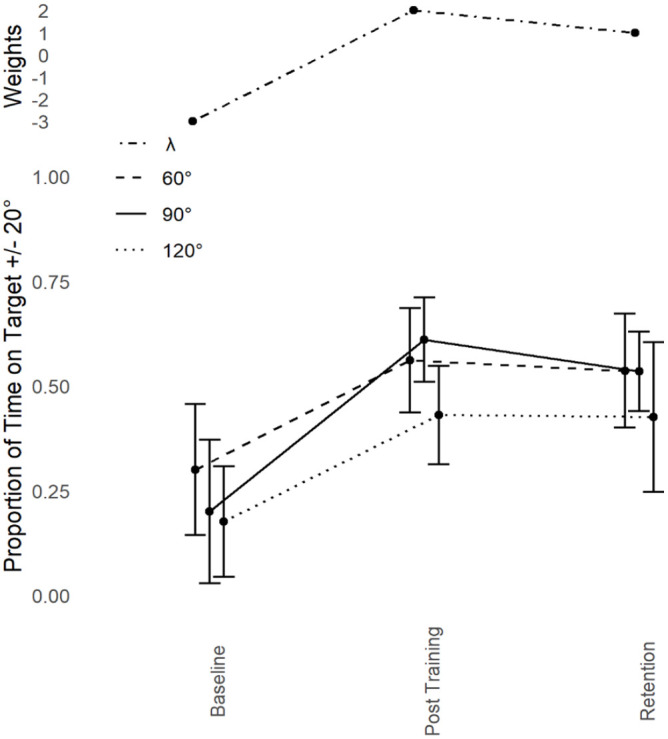
Young adults trained at 90°: Performance transfer. Average performance data (Proportion of Time on Target ±20°, lower) with standard error bars (lower) for the trained phase of 90° and it’s transfer partners 60° and 120° in the three assessment sessions (Baseline, Post-training and Retention) with corresponding Lambda (λ) weights (upper).

Based on Leach et al. (2021b), we predicted transfer to 60° and 120° only. This was expected to be asymmetric in fashion, with 60° showing a greater proportion of transfer than 120°. Refer to Figure 3. In line with predictions, dependent measures contrast analyses revealed significant transfer to 60°, *t*(8) = 5.13, *p* < .001, *g* = 1.71 and 120°, *t*(8) = 6.986, *p* < .001, *g* = 2.32. Both effects were large in magnitude. No transfer was detected at any other phase (0°, 30°, 150°, or 180°, all *p* > .05; Holm–Bonferroni sequential corrections were applied throughout).

Proportion of transfer was calculated across all conditions by taking the difference between Post-training and Baseline performance for the criterion task and dividing that by the difference between the Post-training and Baseline performance for each of the transfer tasks. This assesses both the direction and magnitude of any change in the transfer task, proportional to the changes of the criterion task. Performance at 0°, 150°, and 180° decreased as a function of practice at 90°. This decrease was minimal at 0° (−7%) but more substantial at 180° (−13%) and 150° −21%). There was some increase in performance at 30° (31%), but only substantial increase at 60° (64%) and 120° (62%). The proportion of transfer pattern did not change between Post-training and Retention.

#### Judgement task

Improvement in movement stability at 90° comes with improved perceptual discrimination of 90°, both resulting in a switch of information variable, from relative direction to relative position. To test that this was the case here, we used a contrast analysis to show that the pattern of change in judgement thresholds across Sessions matched that of the action measures, and then we compared judgement performance under the position perturbation with judgements of unperturbed displays.

##### Contrast analyses

**Figure 4.**
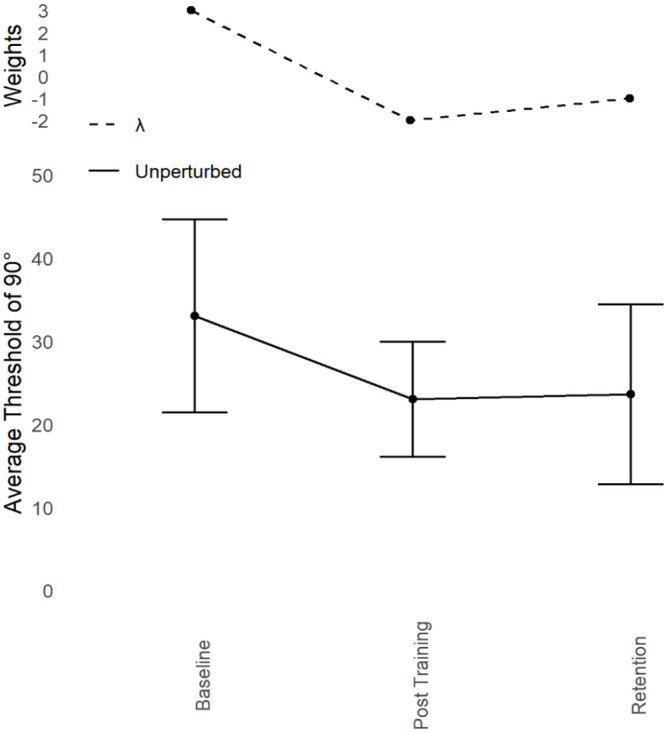
Young adults trained at 90°: Judgement transfer. Average unperturbed Perceptual Judgement Thresholds for 90° (lower) with standard error bars at Baseline, Post-training, and Retention with corresponding Lambda (λ) weights (upper). Participants follow the pattern predicted by reversing the action Lambda weights (3, −2, −1). Participants started out with poor thresholds for perceiving 90°. Perception of 90° improved after training (Post-training) and that improvement showed some depreciation but remained fairly stable (part-retention).

Refer to Figure 4. At Baseline, thresholds for perceiving 90° were high (*M* = 33.03°, *SD* = 11.63°). By Post-training this threshold decreased (*M* = 23.02°, *SD* = 6.93°) and remained relatively low after the Retention period (*M* = 23.61°, *SD* = 10.81°). The λ weights identified in the action learning pattern of 90° were again used to predict the same pattern in the Judgement data, but note that for judgements, “improvement” means a decrease in threshold, so the sign of the λ weights was reversed (3 for Baseline, −2 for Post-training, −1 for Retention).

A dependent measures contrast analysis with the within-subjects factor of Session (3 levels; Baseline, Post-training, and Retention) and the dependent variable of unperturbed judgement thresholds of 90°, revealed a significant effect with a large effect size, *t*(8) = 5.17, *p* < .001, *g* = 1.724. The size of the effect was comparable to Leach et al. (2021b), where *g* = 1.518. YA judgement thresholds at 90° improved in the same way as coordination stability at 90°.

### Unperturbed versus perturbed judgement threshold comparison

**Figure 5.**
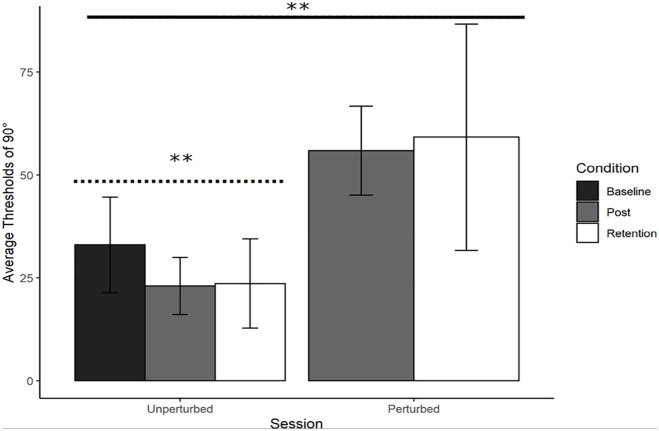
Young adults trained at 90°: Average perceptual judgement thresholds. Average perceptual judgement thresholds for 90° with standard error bars at Baseline, Post-training, and Retention. Significance levels are indicated in the figure (** = *p* < .01). There was a significant main effect of Condition, with the perturbation reducing performance (solid line). The contrast analysis demonstrated that the learning data was a good fit for the unperturbed judgement data (dotted line).

Refer to Figure 5. A repeated measures ANOVA was performed on average judgement thresholds with Session (Post-training and Retention) and Condition (Unperturbed and Perturbed) as factors. There was a significant main effect of Condition, *F*(1, 16) = 29.27, *p* < .001, with no other significant main or interaction effects. Thresholds for identifying 90° were low in the unperturbed condition in both Post-training (*M* = 23.02, *SD* = 6.93) and Retention (*M* = 23.61, *SD* = 10.81) but were much higher in the perturbed condition for both Post-training (*M* = 55.95, *SD* = 10.79) and Retention (*M* = 59.16, *SD* = 27.52). YA participants improved at 90° by switching to using relative position as the information variable.

In summary, this experiment closely replicated the results of Leach et al. (2021b): learning to produce 90° relative phase with coordination feedback transfers to 60° and 120°, improves perceptual judgement thresholds of 90°, and entails switching information variable use from relative direction to relative position.

### OAs

#### Action task

##### Learning

**Figure 6.**
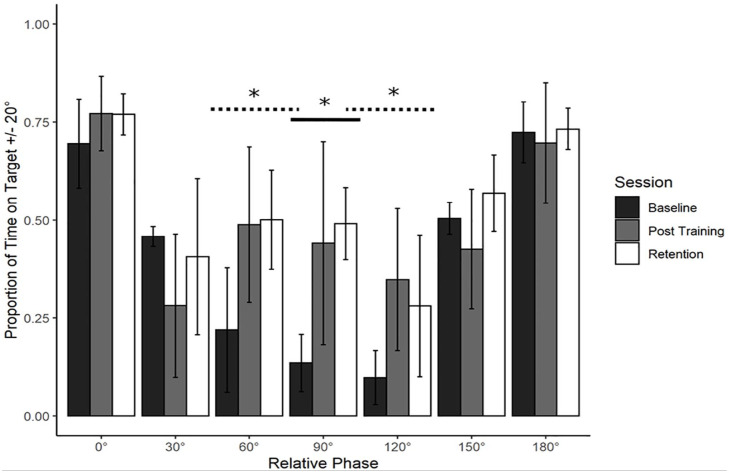
Older adults trained at 90°: Average action data. Average performance data (Proportion of Time on Target ±20°) with standard error bars for all phases in the three assessment sessions (Baseline, Post-training, and Retention). Significance levels are indicated in the figure (* = *p* < .05). There was a significant main effect of Session for the trained phase of 90° (solid line). This learning transferred to 60° and 120° (dotted lines, see “Transfer” section for further details).

Refer to Figure 6. To examine whether and how training at 90° changed performance at 90°, average PTT20 was analysed using a one-way repeated measures ANOVA with Session (Baseline, Post-training, Retention) as a within-subject factor. Descriptive statistics show a difference between Baseline (*M* = 0.13, *SD* = 0.07), Post-training (*M* = 0.44, *SD* = 0.26), and Retention (*M* = 0.49, *SD* = 0.09).

OA participants significantly improved their coordination stability from Baseline to Post-training and that learning was retained after the rest period. There was a significant main effect of Session, *F*(2, 8) = 9.08, *p* < .01. Bonferroni-adjusted post hoc analyses revealed significant differences between Baseline and Post-training *t*(4) = −3.38, *p* < .05, *MD* = −0.31 and Baseline and Retention, *t*(4) = −3.94, *p* < .05, *MD* = −0.36. No other significant comparisons were found. Production of 90° was poor at Baseline, it improved significantly with training and this improvement persisted after the retention period.

##### Transfer

The learning analysis established the pattern of learning and retention at 90° that we will look for at other relative phases, to measure transfer of learning. For the contrast analysis we use here, the λ weights were set at −2 for Baseline, 1 for Post-training and 1 for Retention (as per Rosenthal et al., 2000; recall that the YA pattern was −3, 2, and 1 due to a significant decrease from Post-training to Retention).

**Figure 7.**
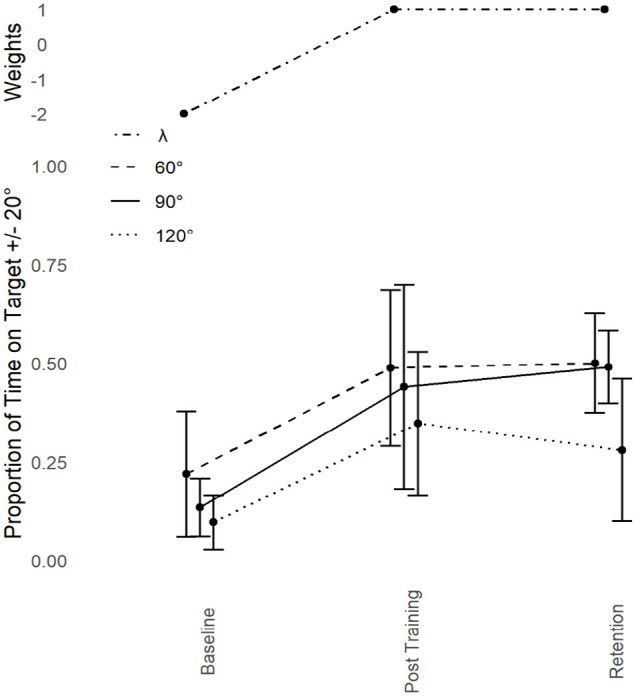
Older adults trained at 90°: Performance transfer. Average performance data (Proportion of Time on Target ±20°, lower) with standard error bars (lower) for the trained phase of 90° and its transfer partners 60° and 120° in the three assessment sessions (Baseline, Post-training, and Retention) with corresponding Lambda (λ) weights.

Based on Leach et al. (2021b) and the YA data, we predicted transfer to 60° and 120° only. This was expected to be asymmetric in fashion, with 60° showing a greater proportion of transfer than 120°. Refer to Figure 7. In line with predictions, dependent measures contrast analyses revealed significant transfer to 60°, *t*(4) = 3.02, *p* = .019, *g* = 1.35, and 120° *t*(4) = 3.064, *p* = .018, *g* = 1.37. Both effects were large in magnitude, but not to the extent seen in the YA data, nor was the magnitude asymmetrical. No transfer was detected at any other phase (0°, 30°, 150°, or 180°, all *p* > .05; Holm–Bonferroni sequential corrections were applied throughout).

Proportion of transfer was calculated across all conditions by taking the difference between Post-training and Baseline performance for the criterion task and dividing that by the difference between the Post-training and Baseline performance for each of the transfer tasks. This assesses both the direction and magnitude of any change in the transfer task, proportional to the changes of the criterion task. Performance at 30°, 150°, and 180° decreased as a function of practice at 90°. This decrease was minimal at 180° (−9%) but more substantial at 150° (−26%) and more substantial yet at 30° (−57%). There was some increase in performance at 0° (25%), but only substantial increase at 60° (88%) and 120° (82%). At Retention the pattern held in all but two places. At 120° the transfer was reduced (51%), while 60° held relatively stable (79%); this is the more commonly observed asymmetric transfer. The negative transfer at 150° was no longer present and became positive (18%). Otherwise the pattern was the same at Post-training.

#### Judgement task

Improvement in movement stability at 90° comes with improved perceptual discrimination of 90°, both resulting in a switch of information variable, from relative direction to relative position. To test that this was the case here, we used a contrast analysis to show that the pattern of change in judgement thresholds across Sessions matched that of the action measures, and then we compared judgement performance under the position perturbation with judgements of unperturbed displays.

##### Contrast analyses

**Figure 8.**
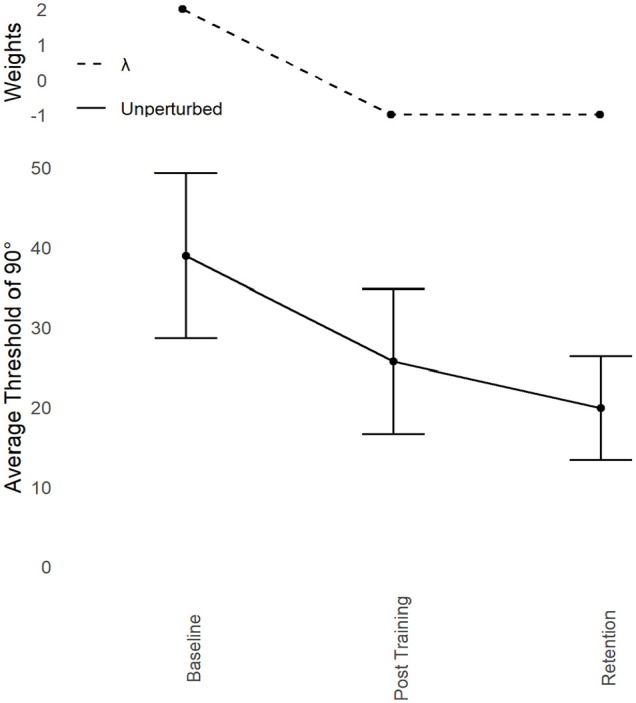
Older adults trained at 90°: Judgement transfer. Average unperturbed Perceptual Judgement Thresholds for 90° (lower) with standard error bars at Baseline, Post-training, and Retention with corresponding Lambda (λ) weights (upper). Participants follow the pattern predicted by reversing the action Lambda weights (2, −1, −1). Participants started out with poor thresholds for perceiving 90°. Perception of 90° improved after training (Post-training) and that improvement was maintained after the Retention period.

Refer to Figure 8. At Baseline, thresholds for perceiving 90° were high (*M* = 38.93°, *SD* = 10.32°). After training, this threshold decreased (*M* = 25.68, *SD* = 9.12) and decreased further after the Retention period (*M* = 19.87°, *SD* = 6.48°). The λ weights identified in the action learning pattern of 90° were again used to predict the same pattern in the Judgement data, but note that for judgements, “improvement” means a decrease in threshold, so the sign of the λ weights was reversed (2 for Baseline, −1 for Post-training, −1 for Retention).

A dependent measures contrast analysis with the within-subjects factor of Session (3 levels; Baseline, Post-training, and Retention) and the dependent variable of unperturbed judgement thresholds of 90°, revealed a significant effect with a large effect size, *t*(4) = 3.25, *p* < .05, *g* = 1.45. OA judgement thresholds at 90° improved in the same way as coordination stability at 90°.

##### Unperturbed vs perturbed judgement threshold comparison

**Figure 9.**
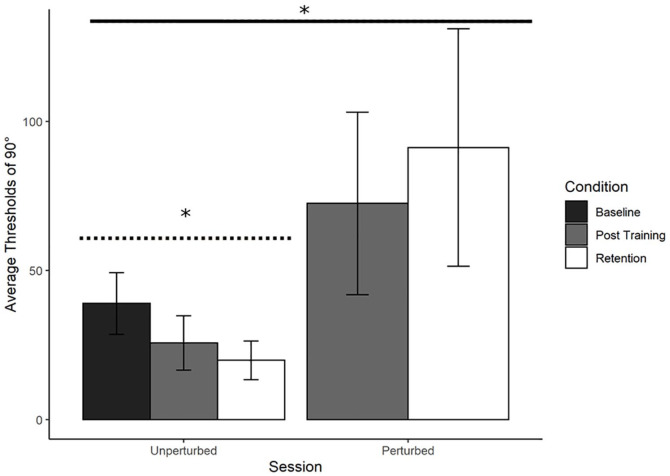
Older adults trained at 90°: Average perceptual judgement thresholds. Average perceptual judgement thresholds for 90° with standard error bars at Baseline, Post-training, and Retention. Significance levels are indicated in the figure (* = *p* < .05). There was a significant main effect of Condition, with the perturbation reducing performance (solid line). The contrast analysis demonstrated that the learning data was a good fit for the unperturbed judgement data (dotted line).

Refer to Figure 9. A repeated measures ANOVA was performed on average judgement thresholds with Session (Post-training and Retention) and Condition (Unperturbed and Perturbed) as factors. There was a significant main effect of Condition, *F*(1,8) = 19.47, *p* < .01, with no other significant main or interaction effects. Thresholds for identifying 90° were low in the unperturbed condition in both Post-training (*M* = 25.68, *SD* = 9.12) and Retention (*M* = 19.86, *SD* = 6.48) but were considerably higher in the perturbed condition for both Post-training (*M* = 72.51, *SD* = 30.6) and Retention (*M* = 91.19, *SD* = 39.82). OA participants improved at 90° by switching to using relative position as the information variable.

In summary, the OA data closely replicated the YA results, as well as Leach et al. (2021b): learning to produce 90° relative phase with coordination feedback transfers to 60° and 120°, improves perceptual judgement thresholds of 90°, and entails switching information variable use from relative direction to relative position. For the first time, we have successfully improved the coordination stability of OAs; we will discuss some potential reasons in the main discussion. There were some differences in the YA and OA performance, however, which we will now lay out.

### YA versus OA comparisons

In terms of overall performance, the YA and OA data were very similar. We ran a mixed ANOVA on the PTT20 data at 90°, with Session (Baseline, Post-training, Retention) as the within-subjects factor and Age (OAs, YAs) as the between subjects-factor. There was a main effect of Session, *F*(2,24) = 45.84, *p* < .01, but no main effect of Age nor an Age × Session interaction. The same was true for the Judgement thresholds; a mixed ANOVA revealed a main effect of Session, *F*(2,24) = 26.43, *p* < .01, but no main effect of Age nor any interaction. Finally, as reported above, the patterns of transfer and response to the position perturbation were all very similar for the two age groups.

Previous studies (Coats et al., 2013, 2015; Ren et al., 2015) also measured the learning rate for YA and OA groups; the “50s Cliff” is a steep decline in the learning rate to around half from the fifties to the sixties. We replicated their analysis here, by fitting an exponential function to the mean 90° performance for each session (Assessment and Training sessions) separately for each age group. The functions were of the form

Equation 1 PTT20=a*exp(−b/S)

where *PTT20* is proportion time-on-task, *S* is Session (1 = Baseline and 10 = Retention), and *a* and *b* are parameters. This yielded an excellent fit for both groups, *r*^2^ = 0.96 (YA) and 0.97 (OA). The values for parameters *a* (with 95% confidence intervals) were: YAs = 0.6599 (0.6216, 0.6983) and OAs = 0.5404 (0.5056, 0.5751). For parameter *b* (again with 95% CIs) they were: YAs = 1.1 (0.8683, 1.331) and OAs = 1.348 (1.073, 1.623).

To evaluate learning rates, the first derivative of the function in Equation 1 was computed as

Equation 2 $$\left(a*b)/S^{2}*exp(-b/S\right)$$

Following equation 2, *a* and *b* are parameters and *S* = Session (1 = Baseline and 10 = Retention). Learning rate is estimated as the derivative evaluated at *S* = 1 as (as per Coats et al., 2014; Ren et al., 2015). The resulting learning rates were YAs = 0.2416 and OAs = 0.1892. These learning rates closely replicate earlier results. Whereby, Coats et al. (2014) found that those in their twenties (0.243) and thirties (0.228) had higher learning rates than those in their fifties (0.203) and sixties (0.125).

The second difference between the age groups was how they progressed through the training sessions. In each training session, the coordination feedback was presented whenever performance was within an error bandwidth of the target relative phase. This error bandwidth was faded over sessions based on performance; if PTT20 was greater than 0.5 in at least 20/30 trials, the error bandwidth was reduced 5° for the next session. Seven of the nine YA participants improved enough to end up in the lowest error bandwidth (±10°), while only one of the OA participants did. The other participants either never progressed (two YA, two OA) or progressed one step in the final session (two OA).

### Exploratory bias analysis

One potential issue is that what we have reported as transfer (say to 60°) could simply be a bias towards one particular instance of the feedback. For example, when presented with the task of moving at 90° an individual might end up moving at 75°, which is within our PTT20 threshold for “on target” for both 90° and 60°. Reducing the analysis bandwidth removes this overlap; if the pattern of results does not change, this confirms the case for transfer.

We repeated the transfer analysis using a bandwidth of ±10°, as per Leach et al. (2021a, 2021b); as in those papers, we report it as a separate exploratory analysis because it was not part of the preregistered analyses. The pattern of transfer results remained identical, just with smaller effect sizes. For YAs, learning only transferred to 60°, *t*(8) = 5.30, *p* < .001, g = 1.77, and 120°, *t*(8) = 6.93, *p* < .001, *g* = 2.31. The same was true for OAs, learning transferred only to 60°, *t*(4) = 3.13, *p* = .017, *g* = 1.40, and 120°, *t*(4) = 2.78, *p* = .025, *g* = 1.24. No other comparisons were significant (0°, 30°, 150°, or 180°, all *p* > .05; Holm–Bonferroni sequential corrections applied throughout).

Overall, as in Leach et al. (2021a, 2021b), the reported results measure actual transfer from 90° to its neighbours, and not bias from actually practicing an intermediate relative phase. Again, this reaffirms the use of PTT20 as a valid and meaningful measure of coordination stability (see also Wilson, Snapp-Childs, & Bingham, 2010, who checked bandwidths of 10°, 15°, and 30° and also showed the bandwidth does not qualitatively affect the results).

## Discussion

Overall, the results of this study confirmed that the change with ageing is a slower learning rate. Given additional time, the OAs were (for the first time) able to reach comparable levels of performance to the younger adults. Otherwise, the learning process was the same: learning to move at 90° for both groups entailed learning to perceive relative phase at 90° using relative position information, and this learning supported transfer of learning to 60° and 120°.

### Implications for model development

The latest version of Bingham’s perception–action model (Herth et al., 2021) explicitly models the learning process at 90°, and the OA data could be readily modelled with a smaller value of the learning rate parameter. However, as yet, this model does not account for the pattern of transfer we first observed in Leach et al. (2021b) and have now replicated in detail here (from 90° to 60° and 120°).

Bingham’s perception–action model is a mechanistic model (Golonka & Wilson, 2019; Wilson, 2022). What this means is that each term explicitly represents a *real part* or *process* that has been empirically confirmed to form part of the complete system (Craver, 2007). Based on the learning studies prior to Leach et al. (2021a, 2021b), Herth et al. (2021) represented relative position as

cos(θ)=xx2˙+x2

When the two oscillators are coupled via this variable, the model yields 90° (with a speed-dependent noise term to model observed variability) and copes well with modelling learning and performing 90°. However, this variable does not yield any other stable relative phases, and so neither does the model, and thus it does not exhibit the same pattern of transfer as humans. The data therefore suggest that at this point, we do not yet have an accurate representation of relative position.

How best to represent relative position is complicated by the results of Leach et al. (2021a). There, we trained participants to produce 60°, which we then confirmed entailed switching to relative position. This transferred to 90° but not 120°; instead we saw transfer to 30°. Learning the same information variable leads to two distinct patterns of transfer! There are two hypotheses as to why:

1. Learning 60° and 90° actually entails learning two different variables that both happen to be susceptible to the position perturbation method. In other words, the position perturbation may not be as specific as Wilson and Bingham (2008) designed it to be, and the model will need to have two different variables added to the mix of options.
2. Something about the training protocol constrains the transfer. The initial feedback error bandwidth is set to +/30°, and so while the participant is learning to differentiate and use relative position, they are, on average, mostly seeing this variable behave within this range. They therefore simply have not had the opportunity to learn that relative position supports stable coordination beyond that range.

Roughly, these two suggestions imply (a) different perceptual learning versus (b) different perception–action learning. At this point, we favour the latter. Wilson and Bingham (2008) performed an ecological task-dynamical analysis of coordinated rhythmic movements to identify what kinematic variables are created by these movements that are also informative about those movements. There are only four candidates: relative direction, relative position, relative frequency, and relative speed, so any additional variable would have to be one of the latter two. Selectively perturbing them, however, simply adds noise to the perception of relative phase (Snapp-Childs et al., 2011; Wilson and Bingham, 2008)—they are not used as information for relative phase. So, at this point, it is not clear what a second variable could be. We recommend that future work here focus on the training protocol; for example, training 90° may not immediately transfer to 30° but it may support savings in learning 30° if the variable used is the same and the problem is lack of experience at 30°.

### Summary

We directly replicated the (then surprising) results of Leach et al. (2021b), by training a group of YAs to move stably at 90° and showing transfer of that learning to 60° and 120°. We then trained a group of OAs as well, and for the first time successfully trained them to produce stable 90°. They also showed the same pattern of transfer, and, as with the younger adults, showed they had learned to move at 90° by switching information variables and perceiving relative phase via relative position. It took the OAs much longer to acquire this skill; however; their learning rate was approximately half that of the younger adults (replicating previous findings). Research on learning in OAs (e.g., about rehabilitation post-stroke) should keep this issue in mind, but note that we had quite high drop-out of participants as well; this extended training works, but is frustrating for the OAs because it takes time to see the results. We suggest factoring this motivation issue into future designs. There remain open questions about exactly how best to model relative position so as to explain how the full range of observed learning and transfer effects emerge from a perception–action dynamic, but there is scope within the task of coordinated rhythmic movement to test our hypotheses.

## Supplemental Material

sj-docx-1-qjp-10.1177_17470218241240983 – Supplemental material for Reduced learning rates but successful learning of a coordinated rhythmic movement by older adults Supplemental material, sj-docx-1-qjp-10.1177_17470218241240983 for Reduced learning rates but successful learning of a coordinated rhythmic movement by older adults by Daniel Leach, Zoe Kolokotroni and Andrew D Wilson in Quarterly Journal of Experimental Psychology

## Notes

1. In practice, this bootstrapping of learning relative position with relative direction needs augmented feedback to work (Wilson, Snapp-Childs, Coats, & Bingham, 2010), although this is not explicitly included in the model.

2. We will come back to this in the “Discussion” section.

3. See the preregistration documents linked in the “Method” section.

4. The Young Adult and Older Adult data collection occurred as two separate studies and the increased payment reflects the fact it was more difficult to recruit older adults for such a long study.
