# Supplementary material for: Reduced learning rates but successful learning of a coordinated rhythmic movement by older adults
Source: Q J Exp Psychol (Hove). 2024 Apr 12;78(3):498–513. doi: 10.1177/17470218241240983 (PMC11874608; doi:10.1177/17470218241240983)
Supplement: sj-docx-1-qjp-10.1177_17470218241240983 – Supplemental material for Reduced learning rates but successful learning of a coordinated rhythmic movement by older adults [file sj-docx-1-qjp-10.1177_17470218241240983.docx]

Supplementary Material for:

# Reduced Learning Rates but Successful Learning of a Coordinated Rhythmic Movement by Older Adults

Daniel Leach,^1^

Zoe Kolokotroni,^1^

&

Andrew D Wilson^1*^

^1^ Psychology, School of Humanities & Social Sciences, Leeds Beckett University, Leeds, UK

* Corresponding Author

Email: [a.d.wilson@leedsbeckett.ac.uk](mailto:a.d.wilson@leedsbeckett.ac.uk%20DrAndrewDWilson@gmail.com) / [DrAndrewDWilson@gmail.com](mailto:DrAndrewDWilson@gmail.com)

Web: <https://cognitioninaction.wordpress.com/>

Twitter: @PsychScientists

Word Count: 7918

This experiment’s design and analysis plan was preregistered (Leach, Kolokotroni, & Wilson, 2019; young adult study, <https://osf.io/x4nef>; older adult study, <https://osf.io/q3k5e>). Data and analysis files are available at <https://osf.io/qvzuh/>.

Supplementary Material 1 – Power Analysis

Two power analyses were performed using G∗Power using the results of Leach et al. (2021b) as input.

## Detecting Very Large Transfer (Input From 60°)

The following is a power analysis performed on the performance data for detecting transfer from 90° to 60°. The analysis is in the form of a one-sided t-test, as is the generation of the t-value from the contrast analysis.

Input: Tail(s) = One

Effect size d = 2.322

α err prob = 0.05

Total sample size = 10

Output: Noncentrality parameter δ = 7.3428087

Critical t = 1.8331129

df = 9

Power (1-β err prob) = 0.9999998

Power to observe very large effects of transfer (d = 2.322) with N = 10 is 1-β of >0.99. This level of power is achieved at N > = 6.

## Detecting Large Transfer (Input From 120°)

The following is a power analysis performed on the performance data for detecting transfer from 90° to 120°.

Input: Tail(s) = One

Effect size d = 1.705

α err prob = 0.05

Total sample size = 10

Output: Noncentrality parameter δ = 5.3916834

Critical t = 1.8331129

df = 9

Power (1-β err prob) = 0.9995092

Power to observe an effect size of d = 1.705 with the above input is 1-β > 0.99. This level of power is achieved at N > = 8.
